# Potential of iPSC-Derived Mesenchymal Stromal Cells for Treating Periodontal Disease

**DOI:** 10.1155/2018/2601945

**Published:** 2018-03-14

**Authors:** K. Hynes, R. Bright, V. Marino, J. Ng, P. J. Verma, S. Gronthos, P. M. Bartold

**Affiliations:** ^1^Department of Dentistry, University of Adelaide, Adelaide, SA 5005, Australia; ^2^South Australian Research & Development Institute, Turretfield Research Centre, Rosedale, SA 5350, Australia; ^3^Mesenchymal Stem Cell Laboratory, Adelaide Medical School, Faculty of Health and Medical Sciences, University of Adelaide, Adelaide, SA 5005, Australia; ^4^South Australian Health and Medical Research Institute, Adelaide, SA 5000, Australia

## Abstract

Mesenchymal stromal cell-like populations have been derived from mouse-induced pluripotent stem cells (miPSC-MSC) with the capability for tissue regeneration. In this study, murine iPSC underwent differentiation towards an MSC-like immunophenotype. Stable miPSC-MSC cultures expressed the MSC-associated markers, CD73, CD105, and Sca-1, but lacked expression of the pluripotency marker, SSEA1, and hematopoietic markers, CD34 and CD45. Functionally, miPSC-MSC exhibited the potential for trilineage differentiation into osteoblasts, adipocytes, and chondrocytes and the capacity to suppress the proliferation of mitogen-activated splenocytes. The efficacy of miPSC-MSC was assessed in an acute inflammation model following systemic or local delivery into mice with subcutaneous implants containing heat-inactivated *P*. *gingivalis*. Histological analysis revealed less inflammatory cellular infiltrate within the sponges in mice treated with miPSC-MSC cells delivered locally rather than systemically. Assessment of proinflammatory cytokines in mouse spleens found that CXCL1 transcripts and protein were reduced in mice treated with miPSC-MSC. In a periodontitis model, mice subjected to oral inoculation with *P*. *gingivalis* revealed less bone tissue destruction and inflammation within the jaws when treated with miPSC-MSC compared to PBS alone. Our results demonstrated that miPSC-MSC derived from iPSC have the capacity to control acute and chronic inflammatory responses associated with the destruction of periodontal tissue. Therefore, miPSC-MSC present a promising novel source of stromal cells which could be used in the treatment of periodontal disease and other inflammatory systemic diseases such as rheumatoid arthritis.

## 1. Introduction

Mesenchymal stromal cells (MSC) are currently being evaluated for their therapeutic efficacy in treating a diverse range of diseases in allogeneic settings without immunosuppressive therapy, due to their immune privileged status and immunomodulatory properties [1]. Moreover, MSC exhibit the ability to home towards and into injured/inflamed sites where they provide therapeutic support through the secretion of anti-inflammatory molecules, cytokines, and trophic factors [2, 3] and through direct cell-cell contact to influence the activities of a range of immune cells [2, 4]. However, issues pertaining to access to MSC and their limited growth potential and associated reduced potency in vitro are major factors restricting the translation of these cells into mainstream treatment approaches [5–7]. In an attempt to overcome issues associated with MSC yield, growth, and potency *in vitro*, numerous groups have attempted to generate MSC-like populations derived from induced pluripotent stem cells (iPSC-MSC) [8–11]. The pluripotent nature of the donor cells opens up the opportunity to generate unlimited quantities of early-passage MSC for clinical use. Importantly, assessment of the tumorigenicity of implanted iPSC-MSC in nonhuman primates failed to identify the formation of any tumors [12].

Assessment of the immunomodulatory properties of iPSC-MSC has shown that they can suppress T-cell proliferation [13–19], modulate the expression of a range of cytokines, promote regulatory T-cell (Treg) expansion, and inhibit the function of both natural killer cells [17, 20] and dendritic cells [14]. Where direct comparisons of the immunomodulatory properties of iPSC-MSC and primary MSC have been reported, iPSC-MSC have proven to be comparable to MSC both *in vitro* and *in vivo* [8, 13, 16, 17, 20–22]. The efficacy of iPSC-MSC has been shown in multiple experimental disease models including, limb ischemia [8], experimental inflammatory bowel disease [15], lupus [14], and autoimmune uveitis [14] and in an autoimmune encephalitis model of multiple sclerosis [21]. Comparative studies reported that iPSC-MSC outperformed BMSC in direct comparisons of their therapeutic efficacy in mouse models of limb ischemia and autoimmune encephalitis-induced multiple sclerosis [8, 21]. The greater therapeutic potential of iPSC-MSC has been attributed to their superior survival and engraftment ability [8, 21]. Therefore, iPSC-MSC are emerging as a highly promising, scalable alternative to current MSC sources for the treatment of a wide range of immune disorders.

Periodontitis is a chronic inflammatory condition of the periodontium, which results from an inflammatory immune response mounted against microbial biofilms on the surface of the teeth. Initiation of the inflammatory immune response is complex involving both innate and acquired immunity. Ultimately, this immune response contributes to the periodontal tissue destruction seen in periodontitis [23]. One study has reported on the effects of tumor necrosis factor alpha-stimulated gene-6 (TSG-6) transduced PSC-MSC in a ligature-based model of periodontitis, providing preliminary evidence that genetically modified iPSC-MSC could serve as an alternative stem-cell-based approach for treating periodontitis [24]. The present study aimed to assess whether iPSC-MSC can inhibit inflammation and bone loss associated with acute and chronic periodontitis.

## 2. Methods and Materials

### 2.1. Animals

Approval for the use of BALB/c mice in this study was obtained from the University of Adelaide, Animal Ethics Committee (Project M-2012-226). The mice were housed in the University of Adelaide PC2 Animal holding facility. Animals were evaluated daily for a number of general health parameters, for example, dull/ruffled coat, a change in temperament, reduced food/water intake, or a reluctance to move, and body weight was recorded. All mice were randomly assigned to either the control or treatment group.

### 2.2. Cell Culture

Mouse iPSC (miPSC) were kindly provided by Professor Paul Verma (Monash University—Faculty of Engineering). The miPSC were generated from tail-tip fibroblasts from NOD/Lt mice using the transcription factors, *Oct4*, *Sox2*, and *Klf4*, in combination with the histone deacetylase inhibitor valproic acid as previously described [25]. Differentiation into MSC-like cells was performed as previously described in the presence of 10 ng/mL of fibroblast growth factor 2 to the MSC media [11, 26].

### 2.3. Flow Cytometric Analysis

Single-cell suspensions were prepared by trypsin digestion for iPSC-MSC cells, or Cell Dissociation Buffer Enzyme-Free phosphate-buffered saline (Thermo Fisher Scientific, MA, USA) to dissociate the iPSC from the mouse embryonic fibroblast feeder layers. Single-cell suspensions were resuspended in blocking buffer consisting of Hank's balanced salt solution (Sigma-Aldrich, MO, USA) supplemented with 5% normal human serum (Australian Red Cross, Melbourne, Australia), 5% FCS (Thermo Fisher Scientific, MA, USA), and 1% bovine serum albumin ((BSA) ICN Biomedical, CA, USA) and incubated on ice for 30 minutes. Approximately, 1 × 10^5^ cells were incubated with specific cell surface marker antibodies reactive with mouse CD34, CD45, CD73, CD105, Sca-1, SSEA1 (BD Biosciences), or isotype control antibodies (10 mg/mL) on ice for 1 hour. After washing, cells were incubated for 30 min on ice with their respective secondary detection antibodies: goat anti-mouse IgG-PE or IgM PE (Southern Biotechnology Associates, AL, USA). After washing, the samples were fixed and then analysed using an Epics XL-MCL flow cytometer (Beckman Coulter, CA, USA). Analysis was performed using the FloExpress software.

### 2.4. Trilineage Differentiation

#### 2.4.1. Osteogenesis

Mineralization was induced as described previously [27]. Briefly, miPSC differentiated to miPSC-MSC-like cells were seeded in triplicate in 24-well plates with 3 × 10^4^ cells/well for imaging and also seeded in six-well plates with 2 × 10^5^ cells/well for RNA collection. Cells were cultured in osteoinductive media (*α*-MEM supplemented with 5% FCS, 100 mM L-ascorbate-2-phosphate, 1 mM sodium pyruvate, 50 *μ*g/mL streptomycin, 50 U/mL penicillin, 2 mM L-glutamine, and dexamethasone 10^−7^ M (Mayne Pharma, NC, USA)) and 1.8 mM inorganic phosphate (KH2PO4; BDH VWR Chemicals, PA, USA) for 28 days. Mineral deposition was identified using Alizarin Red staining (Alizarin Red S; Sigma-Aldrich, MO, USA). Total RNA was extracted from the miPSC-MSC-like cells with TRIzol (Thermo Fisher Scientific, MA, USA), under osteoductive conditions or normal growth medium for 4 weeks.

#### 2.4.2. Chondrogenesis

Chondrogenic induction has been described in detail previously [28]. Briefly, 5 × 10^5^ miPSC-MSC-like cells were centrifuged at 600*g* into cell pellets then cultured in polypropylene tubes in chondrogenic media for 28 days. For histological assessment, the cell pellets were fixed, paraffin embedded, sectioned, stained with hematoxylin and eosin, and immunohistochemically stained with anticollagen type II monoclonal antibody as previously described [28]. Replicate cell pellets were washed then digested with collagenase I (3 mg/mL; Worthington Biochemical, NJ, USA) and dispase II (4 mg/mL; Roche Diagnostics, Basel, Switzerland), then processed for RNA with TRIzol. To assess the level of glycosaminoglycan (GAG) synthesis, 1 × 10^5^ iPSC-MSC-like cells were seeded at per well in 96-well plates. The level of GAG synthesis was measured by ^35^SO_4_ incorporation using a TopCount NXT Microplate Scintillation and Luminescence counter (Perkin Elmer Life and Analytical Sciences) over a 5-day period, normalized to DNA content per well. Quantitation of DNA was performed using the Quant-iTTM PicoGreen dsDNA Assay Kit (Thermo Fisher, MA, USA) as per the manufacturer's instructions, using a Polar Star Optima microplate reader at 540 nm.

#### 2.4.3. Adipogenesis

Adipogenic potential of miPSC-MSC-like cells was assessed as previously described [27, 29]. The miPSC-MSC-like cells (3 × 10^3^) were seeded in 24-well plates then cultured for 28 days in adipogenic induction medium. Lipid deposits were identified with Oil Red O (MP Biomedicals, CA, USA). RNA was isolated from replicate plates, using TRIzol after 28 days of adipogenic induction or under normal growth conditions.

### 2.5. RNA Extraction, cDNA Synthesis, and Real-Time PCR

Total RNA was extracted from cultures using TRIzol (Invitrogen, Grand Island, NY, USA) according to the manufacturer's instructions. Generation of cDNA and real-time analysis was performed as previously described in triplicate [30]. Real-time PCR primers (GeneWorks, Thebarton SA, AUS) used in this study are the following: *BMP-2* (NM_007553.3) fwd 5′-gggacccgctgtcttctagt-3′, rev 5′-tcaactcaaattcgctgaggac-3′; *Opn* (NM_001204201.1) fwd 5′-agcaaactcttgcaagcaa-3′, rev 5′-gattcgtcagattcatccgagt-3′; *Pparγ2* (NM_001127330.2) fwd 5′-ttttccgaagaaccatccgatt-3′, rev 5′-atggcattgtgagacatcccc-3′; *C/ebpα* (NM_001287514.1) fwd 5′-caagaacagcaacgagtaccg-3′, rev 5′-ctcactggtcaactccagcca-3′; *Collagen Type 2α1* (NM_001113515.2) fwd 5′-gcgaccgggagcatataact-3′, rev 5′-gccctaattttcgggcatcc-3′; *Sox9* (NM_011448.4) fwd 5′-cacaagaaagaccaccccga-3′, rev 5′-ggaccctgagattgcccaga-3′; TNF*α* (NM_013693.3) fwd 5′-tgcttgttcctcagcctctt-3′, rev 5′-tgggctacaggcttgtcact-3′; *CXCL1* (NM_008176.3) fwd 5′-aaccgaagtcatagccacac-3′, rev 5-gttggatttgtcactgttcagc-3′; and *β-actin* (NM_007393.5) fwd 5′-ttgctgacaggatgcagaag-3′, rev 5′-aagggtgtaaaacgcagctc-3′.

### 2.6. Inhibition of Splenocyte Proliferation

The miPSC-MSC were inactivated by *γ*-irradiation (30Gy) and plated into a 96-well flat-bottom plate at a concentration of 1 × 10^5^/well 24 hours before the addition of mouse splenocytes prelabelled with 2 *μ*M of carboxyfluorescein diacetate succinimidyl ester ((CFSE) Invitrogen, OR, USA). Splenocytes, from three mouse donors, were cultured in the presence or absence of miPSC-MSC at a 1 : 1 (miPSC-MSC: splenocytes) ratio, in *α*MEM, P/S, sodium pyruvate, L-glut, 10% FCS, and 1 *μ*g/ml of concanavalin A (Con A; Sigma-Aldrich, MA, USA), an inducer of splenocyte proliferation, for 5 days. Colcemid (Thermo Fisher, MA, USA), a cell cycle arresting agent, was used as a positive control at a concentration of 100 ng/ml. Splenocyte proliferation was analysed by flow cytometry to detect green fluorescence (CFSE), and analysis of cell division and proliferation index (average fold expansion) was achieved using FCS 4 express flow cytometry software (De Novo Software, Los Angeles, CA, USA). Proliferation index in cocultures was expressed as a percentage of PBMC proliferation in the absence of immunomodulatory cells. All experiments were performed in triplicate.

## 3. Sponge Model

### 3.1. Heat-Killed *P*. *gingivalis*

The heat-killed *P*. *gingivalis* (HKPG) was prepared by incubating a suspension of these bacteria in sterile PBS with a cell density estimated to be greater than 10^11^ organisms/ml (optical density (600 nm) > 5.0) at 60°C maintained for 10 min. The absence of any viable bacteria was confirmed by culturing a small volume (100 *μ*l) of the heat-killed suspension on a blood agar plate under the anaerobic conditions required for *P*. *gingivalis* growth.

### 3.2. HKPG-Impregnated Sponges

Sterilized polyurethane foam pieces (3 × 2 × 2 mm) were impregnated with HKPG by immersing and compressing the sponges in 1 ml HKPG suspension (10^11^ organisms/ml) and air drying at 37°C under UV light in sterile 12-well plates overnight, to prevent contamination.

### 3.3. Sponge Implantation to Induce Inflammation

All the surgeries were done using the following set protocol: mice were anaesthetized using inhalation anaesthesia by 2% *v*/*v* isoflurane with O_2_ flow rates of 2 litres per minute. Once general anaesthesia was induced, a small 5-6 mm midline dorsal incision was made, followed by blunt dissection to create two subcutaneous pouches in the area of the left and right shoulders into which one HKPG-impregnated sponge was implanted on either side. The wound was closed off with surgical staples, and the animals were placed on a 7-day course of antibiotics to avoid infection and administered pain relief as required. All animals were monitored during recovery, and postoperative observations using clinical record sheets were performed daily. Sponges were left in situ for 21 days and 49 days to assess treatments in the acute and chronic phases of the inflammatory reaction.

### 3.4. Treatment during Inflammatory Stage

To investigate the effect of differentiated MSC-like cells from miPSC on inflammation, the following group treatments were conducted at day 7 postimplantation. Each group contained 6 mice for this study. Groups 1 and 4 PBS controls received PBS via tail vein injection in half the mice, while the other half received PBS via subcutaneous injection into the sponge implant zone. Groups 2 and 5 received tail vein injections of differentiated MSC-like cells, and Groups 3 and 6 received subcutaneous injections into the sponge implant zone. Implanted sponges from groups 1, 2, and 3 were retrieved at day 21 and those in groups 4, 5, and 6 at day 49.

### 3.5. Retrieval Surgery

On the days of retrieval (day 21 and day 49), animals were anaesthetised by inhalation anaesthesia, blood was collected by cardiac puncture, and then the animals were immediately killed by CO_2_ inhalation and cervical dislocation. The sponges were retrieved and cut in half. One-half of the sponge was prepared for histological assessment and the other half for biochemical analysis of cytokines. The spleens were also collected for biochemical analysis and other organs for routine histology.

### 3.6. Histological Preparation and Analysis of Inflamed Sponges

One-half of the sponge from both the left and right flank was placed into 10% phosphate buffered formalin at time of retrieval, kept in formalin at room temperature for two days, washed in PBS, and processed for paraffin embedding. Serial paraffin sections (7 *μ*m) were prepared and stained with haematoxylin and eosin. For each of the mice, one of the sections was chosen at random and evaluated at 10x magnification using a light microscope in order to assign a semiquantitative analysis score for both the severity and infiltration of inflammation. The scoring of the severity of inflammatory changes was based on the percentage of chronic inflammatory cells (predominantly lymphocytes), using a point scale: 0 = normal tissue 0–5% inflammatory cells; 1 = mild inflammation 5–25% inflammatory cells; 2 = moderate inflammation 25–50% inflammatory cells; and 3 = severe inflammation 50% inflammatory cells. Similarly, the scoring system for inflammation infiltration into the sponge was 1 = 0–20% infiltration, 2 = 20–50% infiltration, and 3 ≥ 50% infiltration. The scoring was performed by 2 independent observers who were blinded to the samples. The histological slides were all coded and randomised and then presented to the independent observers for analysis.

### 3.7. Post miPSC-MSC Treatment on Experimental Periodontitis by Oral Inoculation of *P*. *gingivalus*

To further investigate the efficacy of miPSC-MSC-like cells in periodontitis, we performed a pilot study to assess the effects of miPSC-MSC-like cells on a bacterial-induced periodontitis mouse model [31, 32]. Periodontitis was induced in mice through a two-stage inoculation sequence involving the local application of *P*. *gingivalis* to the teeth as well as the systemic administration through oral gavage. Once periodontitis had been established (day 44), half of the mice were treated with an injection, via tail vein of miPSC-MSC-like cells (*n* = 3) whilst the other half received an injection of PBS (*n* = 3). Fourteen days after the administration of miPSC-MSC-like cells, the experiment was completed. Mice underwent micro-CT analysis to assess the CEJ-ABC bone volume. The jaws of the mice were processed for histological analysis. All animal inoculations were performed in a PC2 animal holding facility at the SA Pathology Veterinary Facility.

### 3.8. Live Animal Microcomputed Tomography

Mice were scanned using the Skyscan 1076 high resolution live animal computed tomography (micro-CT) (SkyScan, Bruker, Belgium) as described in detail previously [31]. Measurements were made of any changes in the cementoenamel junction to the alveolar bone crest (CEJ-ABC) length in the jaws, in the mice treated with miPSC-MSC-like cells versus controls. The scanning width was set to 35 mm with a resolution of 9 mm. Animals were anaesthetised prior to scanning with a mixture of ketamine and xylazine and positioned on a polystyrene foam holder then placed within an enclosed container with a HEPA filter at both ends. The container was placed in the 3 cm carbon fibre bed of the micro CT scanner. Mice were scanned three times during the study; preinduction of experimental inflammatory disease to obtain baseline measurements (day 0), post experimental disease induction (day 44), and again at the completion of the study, day 58.

### 3.9. Microcomputed Tomography Data Processing

Scans were reconstructed using SkyScan NRecon software (Version 1.6.6.0). Settings used are smoothing = 1, ring artefact = 15, and beam hardening = 30%, and misalignment compensation was adjusted manually. The BMP files created were opened in CT Analyser (Version 1.12.0.0+) to create a volume of interest. DataViewer (Version 1.5.1.2 64-bit) was used to realign images and save the appropriate plane for the CEJ-ABC distance. Sagittal and coronal images were to determine the CEJ-ABC distance. The images were then opened in CT Analyser to measure CEJ-ABC distance. The histogram settings were set at 100 and 255 to measure CEJ-ABC junction distance. The CEJ-ABC distance was measured between the second and third molars on three slices for each mouse [33].

### 3.10. Histological Scoring for Periodontitis

At day 63, mice were euthanized by CO_2_ inhalation. The heads were fixed in 10% buffered formalin for 48 hours. Following fixation, the specimens were rinsed thoroughly in PBS and then decalcified in 10% EDTA, with agitation for 14 days, replacing the solution regularly over this period. Thorough decalcification was confirmed by radiography, and specimens were paraffin embedded then sectioned at a thickness of 7 *μ*m. For inflammatory scoring and histological analysis, sections were stained with haematoxylin and eosin. Slides were observed using a Leica DM1000 microscope and imaged with a connected Leica DFC450 Camera system (Leica Microsystems, Wetzlar, Germany). The periodontium of the maxillary molars was assessed for inflammation with interdental inflammatory cell infiltrate, change to tissue architecture, papilla reduction, and presence of osteoclasts particularly between the first and second molars. The severity of each parameter was scored on a scale from 0 to 3 (0 = normal, 1 = mild effect, 2 = moderate effect, and 3 = severe effect). The sections assessed for each mouse were all at the level where the distal root of the first molar and proximal root of the second molar were clearly visible [32].

### 3.11. Statistical Analysis

The data collected from this study were analysed by one-way ANOVA. When the global test was statistically significant (*p* < 0.05), post hoc comparisons were performed using the Tukey or Bonferroni's multiple comparison, 95% confidence level. Unpaired *t*-tests (two tail) were also used for 2 group comparisons. All testing was carried out with GraphPad Prism 6 as the statistical analysis package. Significance levels were set at *p* < 0.05 for all tests.

## 4. Results

### 4.1. miPSC-MSC Exhibit a MSC-Like Immunophenotype

**Figure 1:**
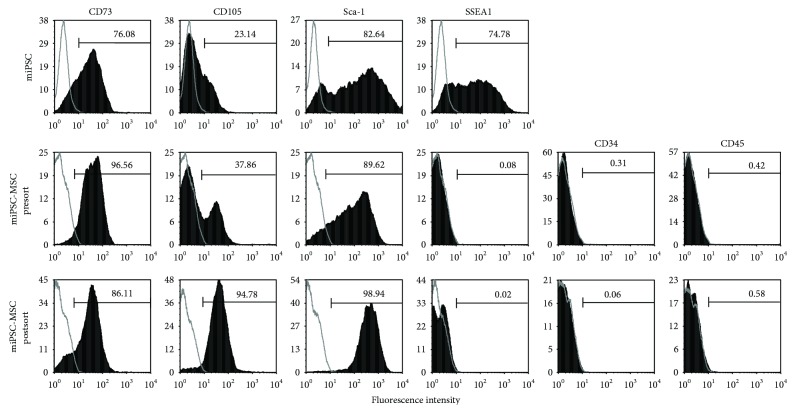
Immunophenotype of the miPSC-MSC. Representative flow cytometry analyses of primary mouse bone marrow MSC (mMSC), noninduced miPSC (miPSC), miPSC induced to differentiate into MSC presorting (miPSC-MSC presort) and after sorting for CD105+, and SSEA1 cells (miPSC-MSC after sort). MSC-related markers CD73, CD105, and Sca-1, pluripotency marker SSEA1, and haematopoietic markers CD34 and CD45 were assessed (solid histogram) relative to their respective isotype controls (open histogram).

Under MSC inductive conditions, the miPSC underwent differentiation towards an MSC-like immunophenotype. Flow cytometric analysis was used to analyse miPSC and miPSC-MSC with respect to MSC-associated markers. Noninduced miPSC were found to express the MSC-associated markers CD73 (76.08%), CD105 (23.14%), and Sca-1 (82.64%) and the pluripotent marker SSEA1 (74.78%) (Figure 1). Following MSC induction, miPSC-MSC displayed high expression levels of CD73 and Sca-1, but lacked expression of SSEA1, CD34, and CD45. However, CD105 was only expressed on a minor subset of miPSC-MSC. Fluorescence-activated cell sorting was subsequently performed to isolate stable CD105 positive expressing miPSC-MSC from the mixed presort population of CD105-positive and CD105-negative cells (Figure 1). Postsort CD105-positive-selected miPSC-MSC were subsequently used in functional experiments described below.

### 4.2. Trilineage Differentiation Potential of miPSC-MSC

**Figure 2:**
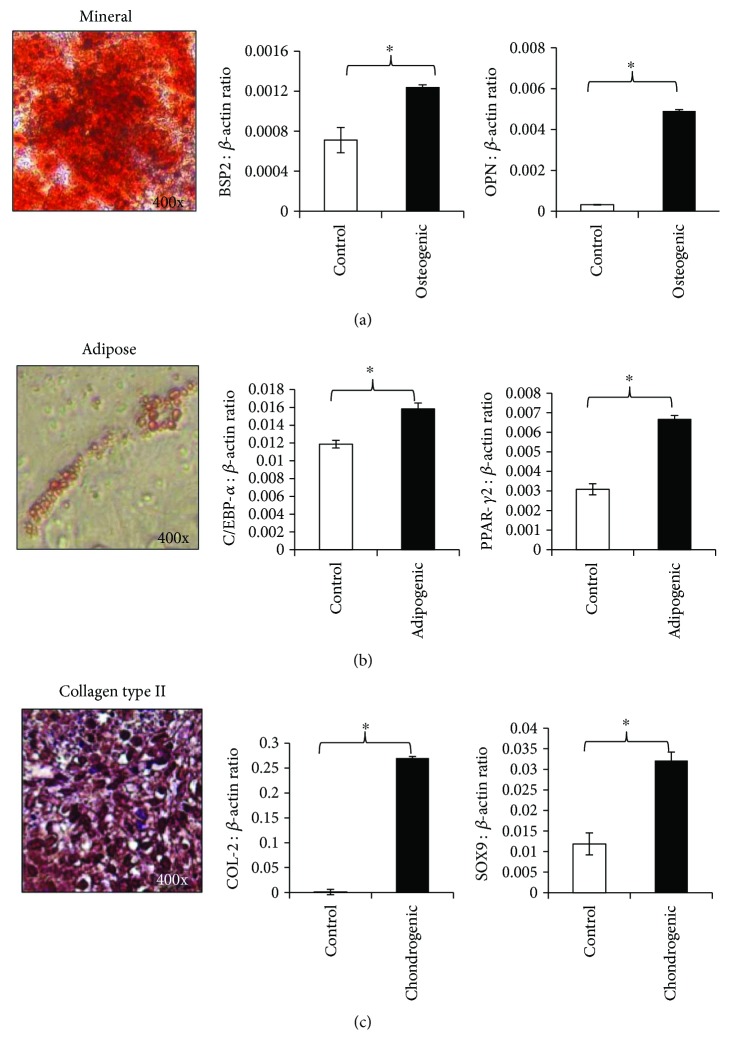
Trilineage differentiation potential of the miPSC-MSC. (a) Representative image of the osteogenic differentiation potential of miPSC-MSC-like cells visualised by Alizarin Red staining of mineralised deposits formed after 4 weeks of culture in osteogenic inductive media. Real-time PCR analysis of the relative expression of genes associated with osteogenesis, bone sialoprotein-2 (BSP2), and osteopontin (OPN). (b) Representative image of the adipogenic potential of miPSC-MSC visualised by Oil Red-O staining for lipid formation after 4 weeks of culture in adipogenic induction medium. Real-time PCR analysis of the relative expression of genes associated with adipogenesis CEBP-*α* and PPAR-*γ*2. (c) Representative image of the chondrogenic differentiation potential of miPSC-MSC visualised by anticollagen type II antibody staining on the chondrocyte pellets formed after culture in chondrogenic media for 4 weeks. Real-time PCR analysis of the relative expression of genes associated with chondrogenesis collagen type II (COL-2) and SOX9. The data represent the mean expression values normalized to the housekeeping gene *β*-actin ± SEM (^∗^*p* < 0.05 unpaired *t*-test).

The ability of miPSC-MSC to undergo trilineage differentiation into osteoblasts, adipocytes, and chondrocytes was assessed *in vitro* to confirm that the cells generated were MSC-like cells. The ability of the miPSC-MSC to undergo osteogenic differentiation was demonstrated by the presence of Alizarin Red-positive-mineralised calcium deposits (Figure 2(a)). Osteogenic induction of miPSC-MSC resulted in a statistically significant increase in expression of the osteogenic-associated genes and bone sialoprotein-2 and osteopontin (Figure 2(a)). The adipogenic potential of the miPSC-MSC was shown by the presence of Oil Red O-positive lipid-containing adipocytes (Figure 2(b)). Adipogenic induction resulted in a statistically significant increase in expression of the adipocyte-associated genes, CCAAT/enhancer-binding protein alpha (C/EBP*α*), and peroxisome proliferator-activated receptor gamma (PPAR-*γ*) (Figure 2(b)). Finally, the chondrogenic potential of the miPSC-MSC was determined in chondrogenic pellets, which formed a collagen type II matrix (Figure 2(c)). Chondrogenic induction led to statistically significant increases in expression of chondrocyte-associated gene collagen type II and SOX9 (Figure 2(c)).

### 4.3. miPSC-MSC Supress Activated Splenocytes In Vitro

**Figure 3:**
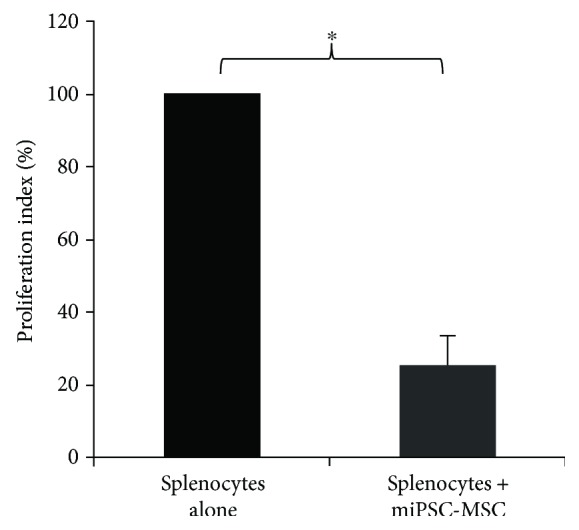
miPSC-MSC mediated suppression of activated splenocytes. Relative proliferation index of the level of proliferation of Con A-stimulated mouse splenocytes (splenocytes alone) compared to splenocytes cocultured with miPSC-MSC as a 1 : 1 ratio. The proliferation level of the splenocytes cocultured with miPSC-MSC was normalised to that of the splenocytes cultured alone. Results show the mean ± SEM data from 3 splenocyte donors performed in triplicate (^∗^*p* < 0.05, unpaired *t*-test).

The immunomodulatory capacity of the miPSC-MSC was assessed following coculture for 5 days with Con A-stimulated mouse splenocytes prelabelled with the fluorescence dye, carboxyfluorescein succinimidyl ester (CFSE), which were then assessed using flow cytometric analysis. In the presence of miPSC-MSC, there was a statistically significant inhibition of proliferating splenocytes in all three donors assessed, with coculture resulting in a significant reduction in proliferation between mitogen-activated splenocytes cultured alone compared to those cocultured with miPSC-MSC (Figure 3).

### 4.4. miPSC-MSC Inhibit Inflammation in an Acute Periodontitis Sponge Model

**Figure 4:**
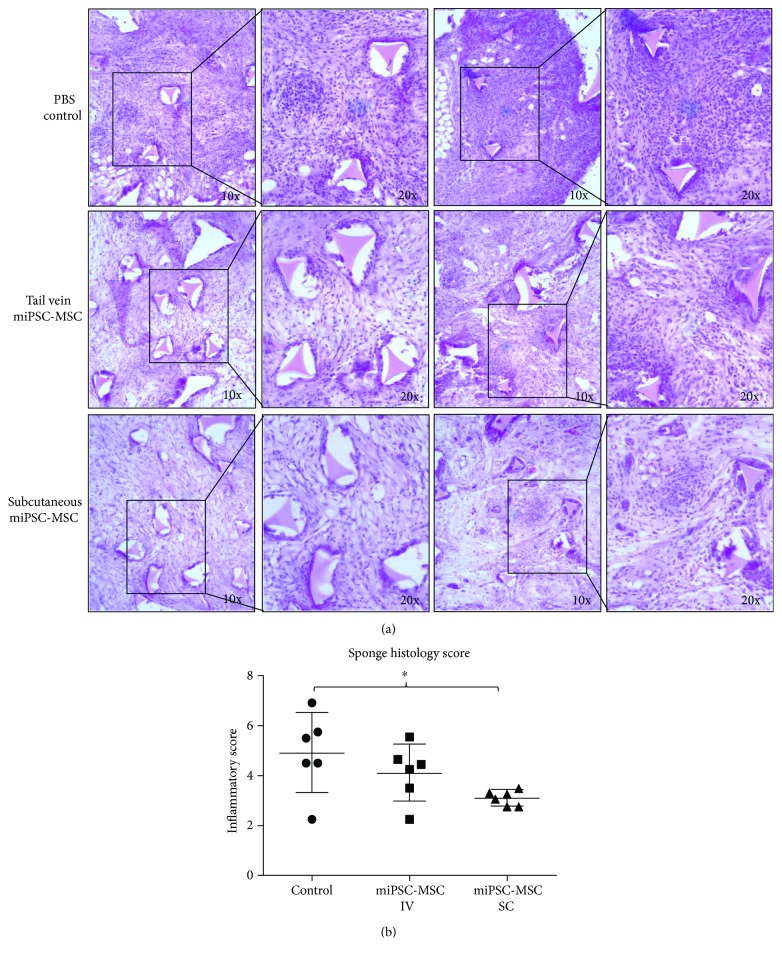
*In vivo* assessment of the immunomodulatory properties of miPSC-MSC in a sponge model of periodontitis. (a) Representative light microscopy images of H&E stained sections of the implanted sponges 14 days after the injection of PBS, or 5 × 10^6^ miPSC-MSC via tail vein injection (IV) or subcutaneous injection (SC). (b) Semiquantitative scoring of the level of immune cell infiltrate present in histological slides of the implanted sponges (^∗^*p* value <0.05 as determined by one-way ANOVA with multiple comparisons, *n* = 6).

To assess the immunomodulatory capacity of the miPSC-MSC in vivo, we employed a sponge model of inflammation to elicit an acute and chronic inflammatory response within the mice. Visual assessment of the histology sections revealed less inflammatory cellular infiltrate within the *P*. *gingivalis* containing implanted sponges in mice treated with miPSC-MSC cells, compared to sponges from control mice which received PBS sham treatment alone (Figure 4(a)). Confirmatory semiquantitative analysis demonstrated a reduction in the inflammatory score in mice, which received miPSC-MSC (Figure 4(b)). The reduction in inflammatory scores achieved through the systemic injection of miPSC-MSC via a tail vein injection of the cells was not statistically significant. Local injection of the miPSC-MSC, via a subcutaneous injection of the cells, resulted in a statistically significant reduction in inflammatory score (Figure 4(b)).

**Figure 5:**
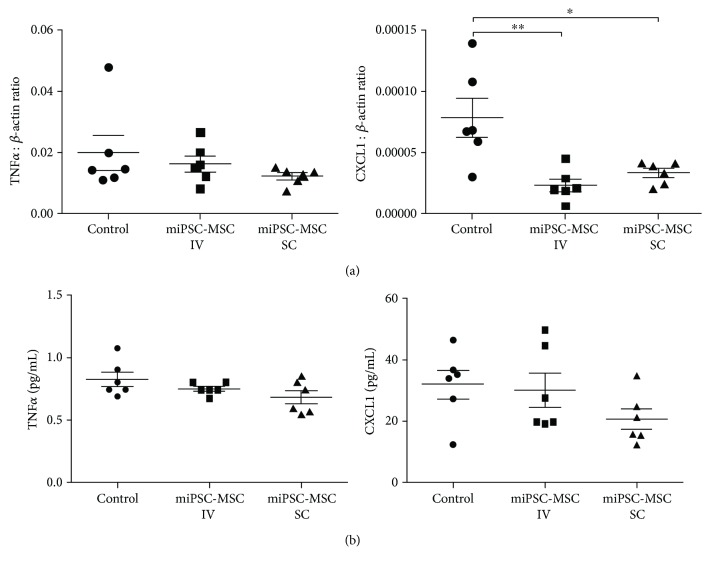
Assessment of cytokine levels in the sponge model of periodontitis. (a) Real-time PCR analysis of the expression of TNF*α* and CXCL1 in the spleens of mice harvested from the sponge periodontitis model. The data represent the mean expression values for each sample normalized to the housekeeping gene *β*-actin ± SEM (^∗^*p* < 0.05 one-way ANOVA with multiple comparisons, *n* = 6). (b) Multiplex analysis of the levels of TNF*α* and CXCL1 present in the blood serum of the mice from the sponge periodontitis model (^∗^*p* < 0.05, ^∗∗^*p* < 0.01, one-way ANOVA with multiple comparisons, *n* = 6).

Gene expression levels of different proinflammatory cytokines were assessed in spleens harvested from mice, by real-time PCR. CXCL1 transcripts were found to be significantly lower in mice, which received a tail vein injection of miPSC-MSC and a subcutaneous injection of miPSC-MSC, in contrast to TNF*α* (Figure 5(a)). Concentrations of CXCL1 protein in blood serum trended towards a lower level in mice injected with miPSC-MSC through both tail vein and subcutaneous injections; however, the decreased levels were not statistically significant compared to the control animals (Figure 5(b)).

### 4.5. miPSC-MSC Supress Inflammation and Reduce Alveolar Bone Loss in a *P*. *gingivalis*-Induced Chronic Periodontitis Mouse Model

**Figure 6:**
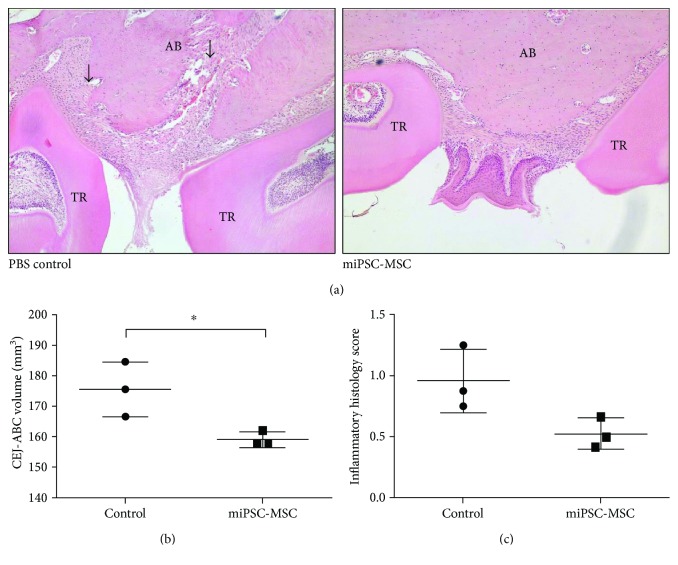
*In vivo* assessment of the immunomodulatory properties of miPSC-MSC in a bacteria-induced periodontitis model. (a) Representative haematoxylin- and eosin-stained sections of the periodontal tissues between two tooth roots showing alveolar bone (AB), tooth root (TR), and periodontal tissue destruction (arrow). (b) Micro-CT analysis of the area between the cementoenamel junction (CJE) and the alveolar bone crest (ABC). (c) Semiquantitative assessment of the inflammatory histology score. Data represent mean ± SD, *n* = 3 (^∗^*p* < 0.05, unpaired *t*-test).

Histological analysis of periodontal structures in mice following oral inoculation with *P*. *gingivalis* indicated less tissue destruction present in the jaws, which received miPSC-MSC treatment compared to PBS alone (Figure 6(a)). Micro-CT analysis confirmed the histological observations, revealing a statistically significant smaller area between the cementoenamel junction (CJE) and the alveolar bone crest (ABC) bone volume in those mice, which received miPSC-MSC when compared to PBS-treated mice (Figure 6(b)). A reduced CEJ-ABC volume is indicative of reduced bone erosion having occurred as a result of periodontitis. Supportive data of inflammatory scores indicated a nonsignificant trend towards reduced inflammation in miPSC-MSC-treated animals (Figure 6(c)).

## 5. Discussion

Our study is a proof-of-concept assessment of whether the conversion of iPSC into iPSC-MSC-like populations could provide a potential alternative stem cell source to primary MSC. We successfully generated miPSC-MSC-like cells, which satisfied the International Society of Cellular Therapy's minimal criteria for defining multipotent MSC [34], based on their plastic adherent properties, expression of key MSC-associated markers, and their ability to undergo trilineage differentiation. However, further genetic profiling analyses are required to determine the equivalence of iPSC-derived MSC-like populations and primary MSC. Other reports have described the differentiation of iPSC into MSC-like populations based on a range of methodologies [10, 11, 35–37] and assessed their potential role in skeletal tissue repair and regenerative medicine applications [38, 39]. One study employed fluorescence-activated cell sorting to purify ESC-derived MSC, based on positive selection using MSC-associated markers and negative selection for pluripotency-associated markers [40]. The present study confirmed that positive immunoselection of iPSC-MSC post conversion, based on CD105 expression, provided stable cultures of CD105-sorted MSC-like populations uniformly expressing MSC-associated markers, while lacking hematopoietic and pluripotent markers. Given the limited life span of ex vivo expanded primary MSC, the optimisation of protocols leading to stable iPSC-MSC preparations may eventually provide an almost unlimited reservoir of MSC for different regenerative medicine applications. However, issue concerning iPSC-MSC stability, safety, production quality, and cost need to be addressed before this technology can be realized.

While regenerative capabilities of iPSC-MSC have been a major focus in studies testing their efficacy in different clinical indications, the immunomodulatory properties of iPSC-MSC have emerged as an exciting new area of investigation into the therapeutic potential of iPSC-MSC for a range of immune/inflammatory-based disease models [13, 19, 22]. The present study confirmed that miPSC-MSC were capable of inhibiting the proliferation of mitogen-stimulated splenocytes. Subsequent experiments were performed to assess the efficacy of iPSC-MSC to inhibit inflammation and bone loss in two established animal models of periodontitis. The first approach assessed the role of miPSC-MSC to inhibit the inflammation in mice implanted with sponges infiltrated with heat-inactivated *P*. *gingivalis* bacteria. Histological assessment revealed reduced trends in the level of inflammatory cellular infiltrate within the sponges harvested from mice, which received miPSC-MSC cells by intravenous injection, seven days after implantation of the *P*. *gingivalis*-infiltrated sponges, compared to the sponges from control mice which received PBS alone. However, local injection of the miPSC-MSC via subcutaneous injection resulted in a statistically significant reduction in inflammatory scores. Mice injected with miPSC-MSC-like cells also showed a decrease in inflammation in the *in vivo* sponge model, with significant decreased gene expression of the immunoregulatory cytokine CXCL1, but not other cytokines such as TNF*α*, TGF*β*, or IL-6 (data not shown). The chemokine, CXCL1, binds to CXCR2 and is expressed by macrophages, neutrophils, and epithelial cells such as gingival fibroblasts [41]. The CXCL1-CXCR2 axis was found to play an important role in mediating neutrophil recruitment in oral mucosa [42]. Moreover, CXCL1-CXCR2 interactions were shown to contribute to periodontal tissue homeostasis [43], where stimulation of human periodontal ligament cells with the inflammation promoter lipopolysaccharide (LPS) increased periodontal ligament cell CXCL1 mRNA expression several fold [44]. Another study, assessing candidate genes associated with periodontitis, identified CXCL1 using an integrative gene ranking method as one of the genes requiring further experimental assessment for its potential role in periodontitis [45].

In a second animal study, we assessed the efficacy of miPSC-MSC intravenous injected into mice with induced chronic periodontitis based on oral inoculation with *P*. *gingivalus*. Micro-CT and histological analyses of the jaw bones identified a statistically significant smaller CEJ-ABC bone volume in those mice which received miPSC-MSC when compared to PBS-treated mice, indicative of reduced bone erosion having occurred as a result of periodontitis. These findings are consistent with the bone regenerative effect of iPSC-derived MSC delivered locally into rats with surgically generated periodontal defects [9]. Collectively, these findings indicate that the reduction of bone loss seen by either systemic or local delivery of miPSC-MSC may be attributed to the modulation of inflammatory responses. In a gene therapy-based study, rat iPSC-MSC were genetically engineered with TNF*α*-stimulated gene-6 (TSG-6), to assess their potential to enhance the therapeutic effects of iPSC-MSC in experimental periodontitis, established by ligature and infection with *P*. *gingivalis* around the maxillary first molar bilaterally [24]. The study found that systemic administration of TSG-6 overexpressing iPSC-MSC was capable of decreasing inflammation in experimental periodontitis and inhibiting alveolar bone resorption, over and above that observed for control iPSC-MSC. TSG-6 has been reported to have an anti-inflammatory effect in several animal models, including arthritis, myocardial infarction, and chemical injury to cornea, and has been attributed to its inhibitory effects on neutrophil migration and plasmin activity [24].

In addition to lymphocyte, neutrophil, and macrophage populations, Tregs have been highly implicated in periodontitis [46], where inhibition of Treg function causes increased numbers of leukocytes within the periodontal tissue and increased bone loss [47]. Furthermore, an imbalance between Th17 and Treg cells has also been suggested to lead to tissue destruction in periodontitis. Reinforcing a role for Th17 and Treg cells in periodontitis is the identification that retinoic acid suppresses experimental periodontitis through modulation of the Th17/Treg imbalance that occurs in periodontitis [48]. Previous reports have shown that iPSC-MSC cells are capable of suppressing T-cell effector populations, Th1/Th2/Th17 cells, and increase levels of Treg cells [13, 18, 22] suggesting a potential role of these cells for the clinical prevention of periodontitis using novel iPSC-MSC therapies [49].

Importantly, conversion of iPSC into MSC-like populations appears to dramatically reduce the potential for tumor formation in vivo. Comparative analyses of the bone-forming capacity of nonhuman primate iPSC and iPSC-derived MSC found no evidence of tumor formation by iPSC-MSC following transplantation in vivo [12]. The findings showed that autologous transplantation of iPSC seeded onto an osteoconductive scaffold resulted in tumor formation and associated inflammation in recipient animals, while iPSC-MSC formed ectopic bone tissue with no associated tumor formation or inflammation. In the present study, histological examination of both periodontitis models found no evidence of tumor formation or increased inflammatory responses. Although we and others have not observed any teratogenic effects of iPSC-MSC in vivo, the safety concerns of genomic instability of iPSC-derived MSC need to be carefully evaluated before clinical translation. In summary, our results demonstrated that miPSC-MSC can be derived from iPSC and that these cells have the capacity to control the chronic inflammatory response that leads to tissue destruction in periodontal disease. Therefore, miPSC-MSC are a promising novel source of stromal cells which could be used in the treatment of periodontitis and other inflammatory systemic diseases such as rheumatoid arthritis, following future advancements in the production of safe and cost-effective iPSC-MSC preparations.

## References

[1] Le Blanc K., Tammik C., Rosendahl K., Zetterberg E., Ringden O. (2003). HLA expression and immunologic properties of differentiated and undifferentiated mesenchymal stem cells. *Experimental Hematology*.

[2] Gebler A., Zabel O., Seliger B. (2012). The immunomodulatory capacity of mesenchymal stem cells. *Trends in Molecular Medicine*.

[3] Wada N., Gronthos S., Bartold P. M. (2013). Immunomodulatory effects of stem cells. *Periodontology 2000*.

[4] Aggarwal S., Pittenger M. F. (2005). Human mesenchymal stem cells modulate allogeneic immune cell responses. *Blood*.

[5] Cakouros D., Isenmann S., Cooper L. (2012). Twist-1 induces Ezh2 recruitment regulating histone methylation along the *Ink4A/Arf* locus in mesenchymal stem cells. *Molecular and Cellular Biology*.

[6] Shi S., Gronthos S., Chen S. (2002). Bone formation by human postnatal bone marrow stromal stem cells is enhanced by telomerase expression. *Nature Biotechnology*.

[7] Wagner W., Ho A. D. (2007). Mesenchymal stem cell preparations—comparing apples and oranges. *Stem Cell Reviews*.

[8] Lian Q., Zhang Y., Zhang J. (2010). Functional mesenchymal stem cells derived from human induced pluripotent stem cells attenuate limb ischemia in mice. *Circulation*.

[9] Hynes K., Menicanin D., Han J. (2013). Mesenchymal stem cells from iPS cells facilitate periodontal regeneration. *Journal of Dental Research*.

[10] Chen Y. S., Pelekanos R. A., Ellis R. L., Horne R., Wolvetang E. J., Fisk N. M. (2012). Small molecule mesengenic induction of human induced pluripotent stem cells to generate mesenchymal stem/stromal cells. *Stem Cells Translational Medicine*.

[11] Hynes K., Menicanin D., Mrozik K., Gronthos S., Bartold P. M. (2014). Generation of functional mesenchymal stem cells from different induced pluripotent stem cell lines. *Stem Cells and Development*.

[12] Hong S. G., Winkler T., Wu C. (2014). Path to the clinic: assessment of iPSC-based cell therapies in vivo in a nonhuman primate model. *Cell Reports*.

[13] Fu Q. L., Chow Y. Y., Sun S. J. (2012). Mesenchymal stem cells derived from human induced pluripotent stem cells modulate T-cell phenotypes in allergic rhinitis. *Allergy*.

[14] Kimbrel E. A., Kouris N. A., Yavanian G. J. (2014). Mesenchymal stem cell population derived from human pluripotent stem cells displays potent immunomodulatory and therapeutic properties. *Stem Cells and Development*.

[15] Sánchez L., Gutierrez-Aranda I., Ligero G. (2011). Enrichment of human ESC-derived multipotent mesenchymal stem cells with immunosuppressive and anti-inflammatory properties capable to protect against experimental inflammatory bowel disease. *Stem Cells*.

[16] Trivedi P., Hematti P. (2008). Derivation and immunological characterization of mesenchymal stromal cells from human embryonic stem cells. *Experimental Hematology*.

[17] Yen B. L., Chang C. J., Liu K. J. (2009). Brief report-human embryonic stem cell-derived mesenchymal progenitors possess strong immunosuppressive effects toward natural killer cells as well as T lymphocytes. *Stem Cells*.

[18] Cheng P. P., Liu X. C., Ma P. F. (2015). iPSC-MSCs combined with low-dose rapamycin induced islet allograft tolerance through suppressing Th1 and enhancing regulatory T-cell differentiation. *Stem Cells and Development*.

[19] Moslem M., Eberle I., Weber I., Henschler R., Cantz T. (2015). Mesenchymal stem/stromal cells derived from induced pluripotent stem cells support CD34^pos^ hematopoietic stem cell propagation and suppress inflammatory reaction. *Stem Cells International*.

[20] Giuliani M., Oudrhiri N., Noman Z. M. (2011). Human mesenchymal stem cells derived from induced pluripotent stem cells down-regulate NK-cell cytolytic machinery. *Blood*.

[21] Wang X., Kimbrel E. A., Ijichi K. (2014). Human ESC-derived MSCs outperform bone marrow MSCs in the treatment of an EAE model of multiple sclerosis. *Stem Cell Reports*.

[22] Ng J., Hynes K., White G. (2016). Immunomodulatory properties of induced pluripotent stem cell-derived mesenchymal cells. *Journal of Cellular Biochemistry*.

[23] Yucel-Lindberg T., Bage T. (2013). Inflammatory mediators in the pathogenesis of periodontitis. *Expert Reviews in Molecular Medicine*.

[24] Yang H., Aprecio R. M., Zhou X. (2014). Therapeutic effect of TSG-6 engineered iPSC-derived MSCs on experimental periodontitis in rats: a pilot study. *PLoS One*.

[25] Liu J., Ashton M. P., Sumer H., O'Bryan M. K., Brodnicki T. C., Verma P. J. (2011). Generation of stable pluripotent stem cells from NOD mouse tail-tip fibroblasts. *Diabetes*.

[26] Hynes K., Menicanin D., Gronthos S., Bartold M. P. (2016). Differentiation of iPSC to mesenchymal stem-like cells and their characterization. *Methods in Molecular Biology*.

[27] Gronthos S., Zannettino A. C., Hay S. J. (2003). Molecular and cellular characterisation of highly purified stromal stem cells derived from human bone marrow. *Journal of Cell Science*.

[28] Isenmann S., Arthur A., Zannettino A. C. W. (2009). TWIST family of basic helix-loop-helix transcription factors mediate human mesenchymal stem cell growth and commitment. *Stem Cells*.

[29] Pittenger M. F., Mackay A. M., Beck S. C. (1999). Multilineage potential of adult human mesenchymal stem cells. *Science*.

[30] Cakouros D., Isenmann S., Hemming S. E. (2015). Novel basic helix–loop–helix transcription factor Hes4 antagonizes the function of Twist-1 to regulate lineage commitment of bone marrow stromal/stem cells. *Stem Cells and Development*.

[31] Cantley M. D., Bartold P. M., Marino V. (2009). The use of live-animal micro-computed tomography to determine the effect of a novel phospholipase A_2_ inhibitor on alveolar bone loss in an *in vivo* mouse model of periodontitis. *Journal of Periodontal Research*.

[32] Cantley M. D., Haynes D. R., Marino V., Bartold P. M. (2011). Pre-existing periodontitis exacerbates experimental arthritis in a mouse model. *Journal of Clinical Periodontology*.

[33] Park C. H., Abramson Z. R., Taba M. (2007). Three-dimensional micro-computed tomographic imaging of alveolar bone in experimental bone loss or repair. *Journal of Periodontology*.

[34] Dominici M., Le Blanc K., Mueller I. (2006). Minimal criteria for defining multipotent mesenchymal stromal cells. The international society for cellular therapy position statement. *Cytotherapy*.

[35] Guzzo R. M., Gibson J., Xu R. H., Lee F. Y., Drissi H. (2013). Efficient differentiation of human iPSC-derived mesenchymal stem cells to chondroprogenitor cells. *Journal of Cellular Biochemistry*.

[36] Liu Y., Goldberg A. J., Dennis J. E., Gronowicz G. A., Kuhn L. T. (2012). One-step derivation of mesenchymal stem cell (MSC)-like cells from human pluripotent stem cells on a fibrillar collagen coating. *PLoS One*.

[37] Villa-Diaz L. G., Brown S. E., Liu Y. (2012). Derivation of mesenchymal stem cells from human induced pluripotent stem cells cultured on synthetic substrates. *Stem Cells*.

[38] Bastami F., Nazeman P., Moslemi H., Rezai Rad M., Sharifi K., Khojasteh A. (2017). Induced pluripotent stem cells as a new getaway for bone tissue engineering: a systematic review. *Cell Proliferation*.

[39] Wu S. M., Hochedlinger K. (2011). Harnessing the potential of induced pluripotent stem cells for regenerative medicine. *Nature Cell Biology*.

[40] Lian Q., Lye E., Suan Yeo K. (2007). Derivation of clinically compliant MSCs from CD105+, CD24- differentiated human ESCs. *Stem Cells*.

[41] Iida N., Grotendorst G. R. (1990). Cloning and sequencing of a new gro transcript from activated human monocytes: expression in leukocytes and wound tissue. *Molecular and Cellular Biology*.

[42] Wu T., Du R., Hong Y., Jia L., Zeng Q., Cheng B. (2013). IL-1 alpha regulates CXCL1, CXCL10 and ICAM1 in network form in oral keratinocytes. *Clinical Laboratory*.

[43] Zenobia C., Luo X. L., Hashim A. (2013). Commensal bacteria-dependent select expression of CXCL2 contributes to periodontal tissue homeostasis. *Cellular Microbiology*.

[44] Nebel D., Svensson D., Arosenius K., Larsson E., Jonsson D., Nilsson B. O. (2015). 1*α*,25-dihydroxyvitamin D3 promotes osteogenic activity and downregulates proinflammatory cytokine expression in human periodontal ligament cells. *Journal of Periodontal Research*.

[45] Zhan Y., Zhang R., Lv H. (2014). Prioritization of candidate genes for periodontitis using multiple computational tools. *Journal of Periodontology*.

[46] Yang J., Wu J., Liu Y. (2014). *Porphyromonas gingivalis* infection reduces regulatory T cells in infected atherosclerosis patients. *PLoS One*.

[47] Garlet G. P., Cardoso C. R., Mariano F. S. (2010). Regulatory T cells attenuate experimental periodontitis progression in mice. *Journal of Clinical Periodontology*.

[48] Wang L., Wang J., Jin Y., Gao H., Lin X. (2014). Oral administration of all-*trans* retinoic acid suppresses experimental periodontitis by modulating the Th17/Treg imbalance. *Journal of Periodontology*.

[49] Gully N., Bright R., Marino V. (2014). *Porphyromonas gingivalis* Peptidylarginine deiminase, a key contributor in the pathogenesis of experimental periodontal disease and experimental arthritis. *PLoS One*.

